# Coordinated recruitment of conserved defense-signaling pathways in PVY^O^-Infected *Nicotiana benthamiana*

**DOI:** 10.1080/15592324.2023.2252972

**Published:** 2023-09-01

**Authors:** Won-Jin Kim, Woong Kim, Youngsoon Kim, Hyeonsook Cheong, Seok-Jun Kim

**Affiliations:** a Department of Integrative Biological Sciences & BK21 FOUR Educational Research Group for Age-Associated Disorder Control Technology, Chosun University, Gwangju, Republic of Korea; b Department of Biomedical Science, Chosun University, Gwangju, Republic of Korea; c Plant Cell Research Institute of BIO-FD&C, Co., Ltd., Incheon, Republic of Korea; d Institute of Well-Aging Medicare, Chosun University, Gwangju, Republic of Korea

**Keywords:** Antioxidant, defense, potyvirus, ROS, ATG4, ATG8

## Abstract

Potato virus Y (PVY) is an aphid-transmitted potyvirus that affects economically important solanaceous species. In this study, the phenomena and mechanisms following infection with PVY were investigated in tobacco (*Nicotiana benthamiana*). In tobacco plants, infection with a mild strain of PVY (PVY^O^) induced stunted growth in the first two leaves at the shoot apex starting 7 days post-infection (dpi), and mosaic symptoms began to appear on newly developing young leaves at 14 dpi. Using enzyme-linked immunosorbent assay and ultrastructure analysis, we confirmed that viral particles accumulated only in the upper developing leaves of infected plants. We analyzed reactive oxygen species (ROS) generation in leaves from the bottom to the top of the plants to investigate whether delayed symptom development in leaves was associated with a defense response to the virus. In addition, the ultrastructural analysis confirmed the increase of ATG4 and ATG8, which are autophagy markers by endoplasmic reticulum (ER) stress, and the expression of genes involved in viral RNA suppression. Overall, our results suggested that viral RNA silencing and induced autophagy may play a role in the inhibition of viral symptom development in host plants in response to PVY^O^ infection.

## 1. Introduction

Potato virus Y (PVY) has a single-stranded positive-sense RNA genome of 9.7 kb^1^ and has recently been reported as the fifth most important plant virus worldwide.^2^ In particular, PVY is known to infect plants by aphids. Potato virus Y also causes serious diseases in important solanaceous crops including tobacco (*Nicotiana tabacum*), potato (*Solanum tuberosum*), tomato (*Solanum lycopersicum*), and pepper (*Capsicum annuum*).^3–5^ The viral disease is especially prevalent worldwide in potato (*Solanum tuberosum*). Potato virus Y infection causes a major loss in potato tuber yield and quality reduction owing to potato tuber necrotic ring spot disease.^3,6^ Therefore, research on plant infections and PVY is ongoing.

Plants rely on a multilayered immune system to fight viral infections. Plant defense mechanisms against viruses include innate immunity, RNA silencing, hypersensitive response, systemic acquired resistance, translational inhibition, ubiquitination and autophagy-mediated proteolysis.^7^ The RNA silencing pathway is a major evolutionarily conserved antiviral mechanism in plants, and viral RNA induces specific plant defense responses involving a large number of plant proteins. Among them, dicer-type double-stranded (dsRNA) and single-stranded (ssRNA) RNases, which belong to the argonaute-type protein family, are assembled in the RNA-induced silencing complex to induce an antiviral response called RNA silencing.^8^ However, plant viruses have adopted various strategies to successfully infect host plants in response to their immune response. The most common strategy used by plant viruses is to produce RNA-silencing virus inhibitors.^9,10^ These inhibitors interfere with the RNA-silencing pathway and affect the antiviral defense of plants.

The antiviral effect on plant viruses suggests a physiological response in plants. A key process by which plant cells respond to stress is vacuolar autophagy, in which cytoplasmic components in the vacuoles are degraded and damaged, and toxic components are removed. During autophagy, cytoplasmic components are non-selectively surrounded within bi-membrane vesicles known as autophagosomes and are degraded in vacuoles/lysosomes for recycling.^11–13^ This degradation process has also been widely conserved throughout evolution, as evidenced by the discovery of autophagy-associated (ATG) genes in all eukaryotes.^14^ In particular, the ATG protein complex and ATG-phosphatidylethanolamine conjugate are essential for autophagosome formation.^15^ In particular, the ATG4 cysteine protease plays an important role in autophagy regulation by promoting covalent binding to phosphatidylethanolamine by processing early ATG8.^16^ In addition, reactive oxygen species (ROS) production, which is increased owing to various stimuli by autophagy, promotes the activation of autophagy to regulate plant development and acclimate to biotic and abiotic stress.^17–19^

Studies on plant resistance mechanisms to PVY have been conducted for a long time to help suppress viruses that infect plants. In particular, we studied tobacco plants sensitive to PVY inoculation because observation of plant symptom development against viruses can be used to analyze plant defense mechanisms and viral pathogenicity. Specifically, we investigated systemic spread of PVY from leaves above infected leaves of tobacco plants from the time of PVY infection until the onset of severe mosaicism. Based on these results, the rate and severity of whole-plant infection according to PVY infection were confirmed, and the antiviral effect during virus recognition was confirmed through the effects of ROS and autophagosome production, intracellular response, and RNA silencing.^10,20^

Based on an understanding of the molecular mechanism of the antiviral effect in tobacco plants, we propose that the regulation of autophagy and RNA-silencing pathways is an effective means of controlling PVY infection in the early stages of tobacco plant development.

## 2. Materials and methods

### 2.1. Plant materials and growth conditions

*Nicotiana benthamiana* seeds were germinated in a growth room at 24 ± 2°C under a 16 h light/8 h dark photoperiod. The plants were then incubated in pots for 4 weeks and then used for PVY^O^ inoculation.

### 2.2. PVY^O^ inoculation and sample collection

Potato virus Y^O^ (PV-575) was obtained from the American Type Culture Collection (ATCC). The virus inoculum was prepared using 100 mg of silicon carbide in 3 mL of 50 mM potassium phosphate buffer (pH 7.5 by grinding 100 mg of PVY^O^-infected leaves. Four-week-old *N. benthamiana* plants were infected with PVY^O^ on their first leaf using cotton swabs. After infection, plants were incubated overnight in the dark in a humid box. Un-infected plants were incubated under the same conditions. Leaves of PVY^O^-infected plants were harvested at 0, 3, 7, 9, 11, 14, and 21 days post-inoculation (dpi). All the samples were immediately frozen in liquid nitrogen and stored at − 70°C. In addition, the length of the leaves, excluding petioles, was measured.

### 2.3. Enzyme-linked immunosorbent assay (ELISA)

To measure the accumulation of viral particles in PVY^O^-infected plants, it was performed by ELISA Reagent Set for Potato virus Y (PVY) (Agdia, Elkhart, IN, USA). First, the leaves of the infected plants were ground with Agdia general extract buffer at a ratio of 1:10 (FWg/mL). The prepared extracts (100 μL each) were added to an anti-PVY^O^-coated micro-plate. The cells were then incubated overnight at 4°C. The wells were washed five times with PBS-T. The wells were then washed eight times with PBS-T, followed by adding 100 μL of enzyme-conjugate buffer to each well and incubating for 2 h at room temperature in the dark. The wells were then washed with PBS-T, PNP substrate buffer (100 μL) was added to each well and incubated for 1 h in a humid box under dark conditions. Absorbance was measured at 405 nm using a UV spectrophotometer (Biotek, Winooski, VT, USA).

### 2.4. RNA extraction, cDNA synthesis, and reverse-transcription PCR (RT-PCR)

After PVY^O^ inoculation, RNA was extracted from the 3^rd^, 7^th^, and 9^th^ leaves harvested from 11 and 14 dpi plants. To extract RNA, leaves were ground with liquid nitrogen. RNA was extracted by adding 1 mL of Ribo Ex™ (GeneAll, Seoul, Korea) per 100 mg of ground leaves. The purified RNA was quantified using a UV-spectrophotometer, and 2 μg of each sample was synthesized as cDNA using reverse transcriptase (Hyperscript^TM^, GeneAll). The expression of genes related to autophagy and other processes was confirmed using RT-PCR. Primer sequences are listed in Supplementary Table 1.

### 2.5. Superoxide radical staining

Nitroblue tetrazolium (NBT) (Sigma-Aldrich, Burlington, MO, USA) staining was performed for the detection of superoxide radicals. 1 dpi above leaves (1, 2, 3, 4, 5 and 6) and 7 dpi above leaves (1, 3, 5, 7, 8 and 9) were harvested from 4-week-old plants. After that, the leaves were stained over night in the dark by 0.2% NBT staining solution {NBT 2 mg/ml in sodium phosphate buffer (pH 7.5) 50 mM}. After overnight, the NBT staining solution was removed, and destained at 70°C for 20 mins with a destaining solution (acetic acid 1: glycerol 1: EtOH 3). The level of ROS in stained leaves was observed by NBT staining.

### 2.6. Morphological length measurement of leaves

4-week-old tobacco plants were infected with PVY by inoculation. Then, the length of leaves of un-infected and PVY-infected plants were measured with Vernier Calipers from 4 dpi to 14 dpi. To compare the length of the leaves, 4 plants were measured repeatedly.

### 2.7. Western blot analysis

Frozen tobacco leaves were ground in liquid nitrogen. The lysate was resuspended by adding two volumes of lysis buffer consisting of 50 mM sodium phosphate (pH 7.0), 10 mM EDTA (pH 8.0), 0.1% Triton X-100, 0.1% sodium dodecyl sulfate, 200 µM phenyl methanesulfonyl fluoride, 250 mM sucrose, 10% glycerol and protein inhibitor cocktail (Sigma Aldrich), 4 mM dithiothreitol, and 10 mM 2-mercaptoethanol. The lysate was incubated by shaking at 4°C for 2 h and then centrifuged at 13,200 rpm for 20 min (4°C). The extracted protein supernatant was subjected to a Bradford assay for protein quantification. Protein samples (20 μg) were separated by 8–12% sodium dodecyl sulfate-polyacrylamide gel electrophoresis and transferred to a polyvinylidene fluoride (Immobilon-FL; Merck Millipore Ltd.) membrane. The membranes were blocked with TBS-T containing 5% skim milk for 1 h, washed, and incubated with the primary antibody overnight. The membrane was then incubated with secondary antibody for 2 h. The membranes were detected using enhanced chemiluminescence (Claro Sola; BioD Co., Ltd., Korea). Antibody information is presented in Supplementary Table 2.

### 2.8. Immunohistochemistry

For histological observation of PVY infection in tobacco, the 8^th^, 10^th^ and top stems of the control and infected plants were harvested 14 dpi. Stems of the control and infected plants were fixed in 4% paraformaldehyde in 50 mM sodium phosphate buffer (pH 7.0). The fixed specimens were embedded in paraffin and cross-sectioned. Then, paraffin was removed with xylene and hydrated with Ethanol 100, 90, 80, 70, 50 and 30% sequentially. Tissues were then incubated for 10 min using 0.1% PBT (0.1% Triton X-100). Then, PBS washing was performed for 5 mins x 2 times, followed by blocking with 3% BSA for 1 h at RT. The PVY-Vn and ATG4 primary antibody was incubated in the plant tissue, and goat anti-rabbit IgG rhodamine red X was attached for fluorescence observation.

### 2.9. Transmission electron microscopy analysis

To observe the ultrastructure of the virus-infected plant tissue, glutaraldehyde-fixed specimens were prepared. The samples were post-fixed in 1% osmium tetroxide (O_s_O_4_) in 100 mM sodium phosphate buffer (pH 7.0). The fixed samples were dehydrated in increasing concentrations of ethanol (30, 50, 70, 80, 90, and 100%) for 15 min. The dehydrated samples were infiltrated with propylene oxide and embedded in Epon 812 (Electron Microscopy Sciences, PA). The Epon blocks were cut into semi-thin (1 μm) and thin (100 nm) sections. Semi-thin tissue sections were stained with 1% toluidine blue. Thin tissue sections were stained with 4% uranyl acetate. Tissue sections were observed at 50 kV using a JEM-2000 FⅫ electron microscope (JEOL Ltd., Tokyo, Japan) at the Chonnam National University Hospital.

### 2.10. Statistical analysis

Statistical analyses were carried out using the *t*-test to evaluate whether the means were significantly different using Prism 5.0 (GraphPad Software, San Diego, CA, USA). Statistical significance was set at *P* < 0.05.

## 3. Results

### 3.1. Developmental stage-dependent growth of PVY^O^-infected plants

**Figure 1.**
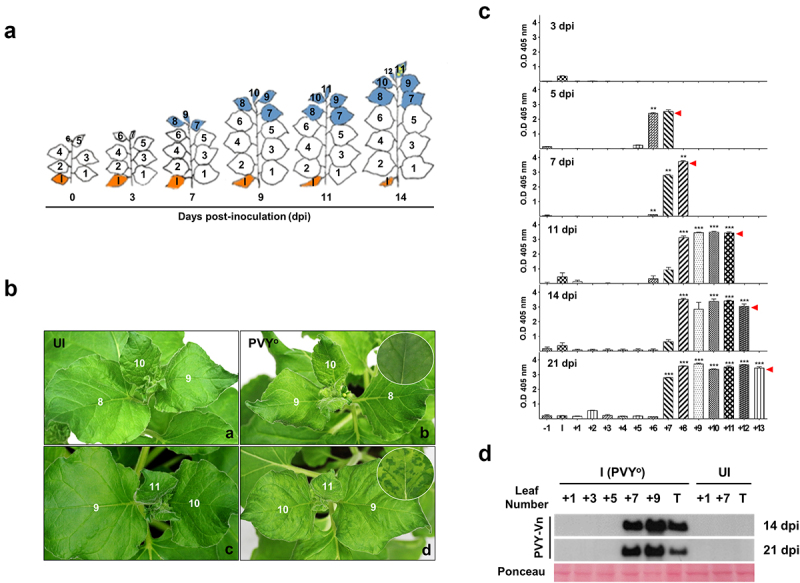
Symptom development and virus accumulation in *Nicotiana benthamiana* infected with potato virus Y-O (PVY^O^). (a) Developmental stages observed during systemic infection of *N. benthamiana* plants with PVY^O^. Control, un-infected 4-week-old plant; PVY^O^, plants infected with PVY^O^. Numbers represent the leaf number above the inoculated leaf (I) on the base. Orange indicates inoculated leaves. Blue indicates that virus protein is detected in the leaves. Mosaic symptom is illustrated with yellow dots on the leaf. (b) disease symptoms of PVY^O^ appeared in a systemic leaf at the top of the *N. benthamiana* plant at 14 days post-infection (dpi). a, control plant at 9 dpi; b, infected plant at 9 dpi; c, control plant at 14 dpi; d, infected plant at 14 dpi. (c) Relative amount of virus in the leaves of *N. benthamiana* infected with PVY^O^. Virus content was measured in the leaves of inoculated plants using ELISA. The Y axis indicates ELISA absorbance values. Numbers represent leaf number above the inoculated leaf (l). Data are presented as mean ± SEM (*n* = 4). Red arrows display location of the top leaf at the indicated day. (d) PVY coat protein (CP) accumulation in *N. benthamiana* plants infected with PVY^O^. Western blot analysis of the PVY coat protein (CP) accumulation in infected *N. benthamiana* plants. Lanes are as follows: the systemic leaves of infected plants (I) and un-inoculated healthy plants (UI). Numbers represent the leaf number. Top leaf (T).

When 4-week-old plants had five leaves (small 6^th^ leaf), the second leaf at the base of the plant was mechanically inoculated with PVY^O^. The growth of the infected plants was monitored until the end of the plant lifecycle (data not shown). The experiment focused on the resistance response against virus spreading from the basal leaf to the top of the PVY^O^-infected plants for up to 21 days. The virus spread rapidly to the upper leaf of the infected plant; however, mosaic symptoms appeared at 14 dpi (Figure 1a). The symptoms of infection included leaf curl, unstable growth, and mosaic symptoms. In particular, symptoms appeared in newly growing leaves (Figure 1b).

Leaf length was measured at 4 dpi to examine growth retardation in the growing leaves. Leaf length was measured from the 6^th^ leaf to the apex using a ruler, and the length was measured up to 14 dpi. (Supplementary Fig. S1). The length of the leaves that had already grown (6^th^ leaf) at inoculation was almost the same as that of the un-infected control. However, the growth rate of new leaves was inhibited over time compared to that of the control (7^th^ leaf or more). At 14 dpi, growth inhibition was observed in the infected leaves as follows: 4.3%, 15.7%, 17.1%, 25.4%, 54.5%,35.1%, and 36.8% in the 6^th^, 7^th^, 8^th^, 9^th^, 10^th^, 11^th^, and 12^th^ leaves, respectively.

### 3.2. Detection of PVY coat protein in young leaves of infected plants

Viral particles were quantified using ELISA to examine the correlation between the inhibition of leaf growth and viral infection. All leaves were monitored for the accumulation of viral particles in plants at 3, 5, 7, 11, 14, and 21 dpi (Figure 1c). Only a negligible amount of viral particles was detected in the inoculated leaves until 3 dpi. However, from 5 dpi, a large number of viral particles were produced in the upper part of the plant. Although the number of leaves increased as the plants grew, viral particles were distributed around the newly formed leaves in the upper part. At 14 dpi, not only did viral particles accumulate in the upper leaves, but a small amount of viral particles was also detected in the lower leaves of the plant (Figure 1c).

To verify the distribution of viruses in infected plants, western blotting was performed to observe the location and amount of viruses on the leaves of infected plants at 14 and 21 dpi (Figure 1d). A large amount of viral coat protein accumulation was observed in the upper part of the plants. Viral coat protein accumulation was prominently observed in newly formed leaves, whereas the virus spread over time after infection. Immunolocalization was performed to observe the intercellular localization of the virus in the infected plants. Viral coat proteins were distributed around the vascular bundles and were also detected in the outer layer of the cortical cells in the stems of 14 dpi plants. The amount of virus was higher in the 10^th^ leaf than in the 8^th^ leaf (Supplementary Fig. S2).

### 3.3. Anatomical changes observed in systemic leaves after PVY^O^ infection

To explain the growth retardation of leaves during the spread of PVY^O^, the 8^th^ and 10^th^ leaves of 9 and 14 dpi plants were collected. Semi-thin sections of the upper epidermal cells, palisade parenchyma, and vascular bundles were observed within the spongy parenchymal layer (Supplementary Fig. S3). When the 8^th^ leaf at 9 dpi was compared to leaves of healthy plants, the number of cells in the spongy parenchyma layer of the infected plant leaves was reduced compared to those in normal plants. A distinct change was observed in the 10^th^ leaf at 14 dpi. Compared to the infected plants, the un-infected cells were small and compact, and the arrangement of the cells was irregular owing to massive cell division. In addition, the decreased number of cells inside the leaf and the slow growth of infected leaves were reflected in the difference in the leaf thickness of infected plants.

### 3.4. ROS production in tobacco plants after PVY^O^ viral infection

**Figure 2.**
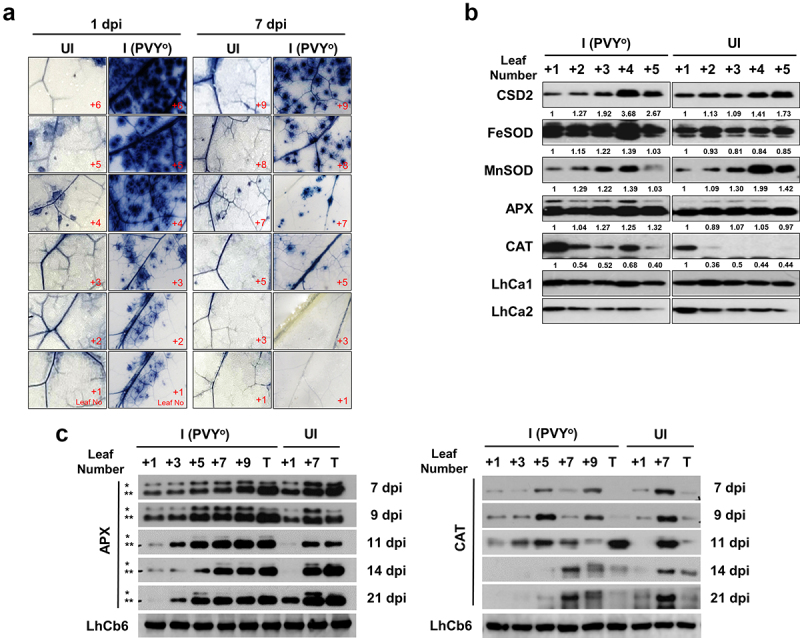
Superoxide generation and induction of ROS-detoxifying enzymes in the leaves of virus-infected plants. (a) detection of superoxide accumulation in leaves of *Nicotiana benthamiana* infected with potato virus Y-O (PVY^O^). The numbers indicate the order of systemic leaves above the infected leaf at indicated day {1 and 7 days post-infection (dpi)}. (b) effect of PVY^O^ on antioxidant enzyme accumulation in systemic leaves of *N. benthamiana* at 3 h after infection. The numbers represent the order of systemic leaves above the infected leaf at 3 h post-inoculation (hpi). Expression levels of antioxidant enzymes. Protein extracts were prepared from the leaves for western blot analysis to determine the levels of protein accumulation. I, leaves infected with PVY^O^; UI, un-infected healthy leaves as control. (c) effect of PVY^O^ on antioxidant enzymes in systemic leaves of *N. benthamiana* infected with PVY^O^. The numbers represent the order of systemic leaves above the infected leaf at the indicated time. Top leaf (T), Right panel: CAT, catalase; left panel: APX, ascorbate peroxidase. Protein extracts were prepared from the leaves for western blot analysis to determine the levels of protein accumulation. I, leaves infected with PVY^O^; UI, un-infected healthy leaves as control. Asterisk in left panel indicates peroxisomal (*) and cytoplasmic (**) APX.

When a plant is infected with a pathogen, the production of reactive oxygen species (ROS) is one of the earliest cellular responses following successful pathogen recognition. In this study, we investigated whether infection of tobacco plants with PVY^O^ leads to the generation of superoxide, which is followed by the production of hydrogen peroxide, resulting in the activation of defense mechanisms. After PVY^O^ infection, leaves at 1 and 7 dpi were stained with nitro blue tetrazolium. The results showed that virus inoculation caused severe superoxide generation in whole leaves (Figure 2a). As shown in Figure 2a, histochemical detection of superoxide showed intense blue staining of the cell spots after viral infection. In contrast, nitro blue tetrazolium precipitation was not visible in the leaf blades of the un-infected plants. Generation of superoxide was dramatically induced in the plant 3 h after viral infection (Figure 2b). Notably, ROS were generated mainly along the veins of leaves. The amount of ROS decreased in the lower leaves over time after PVY^O^ infection. However, intense amounts of ROS were still detected in the upper leaves.

### 3.5. Induction of antioxidant enzymes in the leaves of tobacco after viral infection

Under viral infection, the ROS levels were dramatically increased compared with those in healthy plants. Thus, the expression levels of ROS scavenging-related proteins were examined in the leaves of infected plants (Figure 2b). The experiments focused on the changes in the levels of two chloroplast superoxide dismutases (SODs) (CSD2 and FeSOD) and a mitochondrial SOD (MnSOD) in tobacco leaves from the base to the apex at 3 h after infection. The steady-state protein level of chloroplast SODs was highly enhanced for efficient detoxification of superoxide in response to viral infection compared to that of un-infected control plants. MnSOD levels barely changed in response to viral infection. Notably, ascorbate peroxidase (APX) levels consistently increased from the leaf position to the apex of infected plants in response to biotic stress. These results indicate that superoxide generation occurred mainly in the peroxisome and cytoplasm.

Additionally, to perform a systemic analysis of H_2_O_2_ scavenging enzymes in response to viral infection, APX and catalase expression levels were evaluated in infected tobacco plants at 7, 9, 11, 14, and 21 dpi. Ascorbate peroxidase expression levels were found to be significantly increased until 9 dpi, whereas the overall expression levels decreased from 11 dpi. However, enhanced catalase expression was observed in the systemic leaves at 11 dpi, which may be induced along with mosaic symptom development in the young leaf at the apex of the plant (Figure 2c). However, the expression level decreased at 14 dpi, which was when the symptoms appeared. In general, the expression of APX and catalase decreased over time in lower leaves. In addition, Lhca1 showed similar expression levels in the systemic leaves compared to un-infected control plants at 3 h after infection, whereas Lhca2 expression was slightly reduced in the plant (Figure 2b).

ROS accumulation may play an important role in the regulation of antioxidant gene expression under biotic stress conditions. These results showed that the expression of CSD2, FeSOD, and APX was highly upregulated in response to cellular ROS levels. Upon virus attack, ROS, including superoxide and H_2_O_2_, can help activate defense reactions in infected cells or serve as a signal to activate defense responses in distant un-infected tissues.

### 3.6. Ultrastructural changes in the cell of PVY^O^-infected leaves

**Figure 3.**
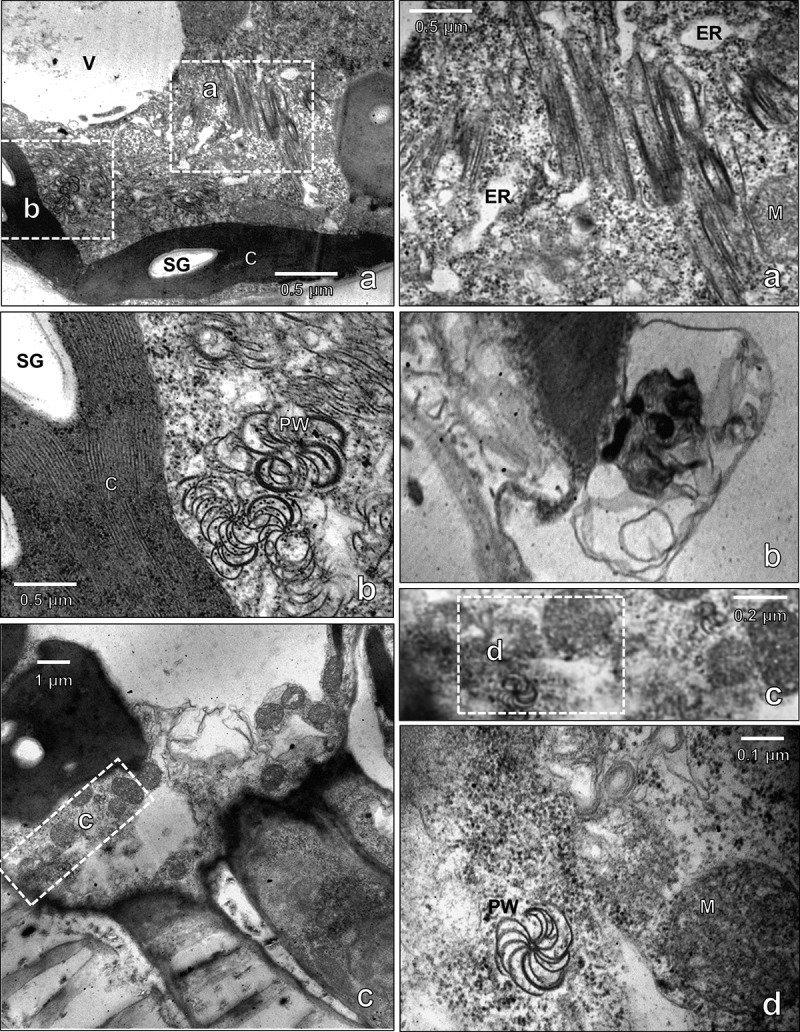
Transmission electron micrographs of cytoplasmic inclusions in the cytoplasm of potato virus Y-O (PVY^O^)-infected tobacco. Tobacco (*Nicotiana benthamiana*) were mechanically inoculated with the PVY^O^ and the 8^th^ leaf above the inoculated leaf was prepared for electron microscopy at 9 days post-infection (dpi). (a) pinwheel (pw) and laminated aggregate (a) cytoplasmic inclusions are clearly visible in the cytoplasm of PVY^O^-infected tobacco leaves. (b) PVY^o^’s inclusion body observed in cytoplasm (b) autophagosome (c) pinwheel in phloem. Vacuole (V), starch grain (SG), chloroplast (c), endoplasmic reticulum (ER), mitochondria (M). Scale bars represent as indicated.

**Figure 4.**
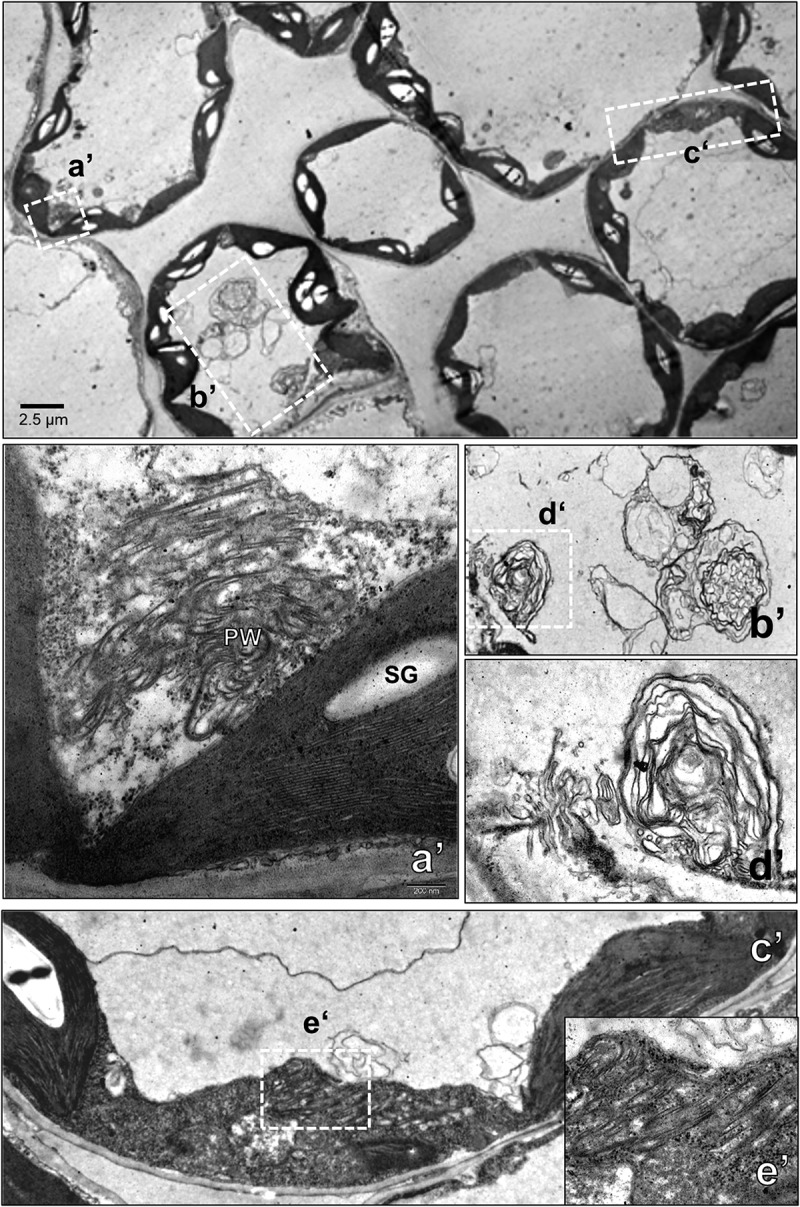
Transmission electron micrographs of cytoplasmic inclusions in chloroplasts of potato virus Y-O (PVY^O^)-infected tobacco. The 10^th^ leaf above the inoculated leaf was prepared for electron microscopy at 9 days post-infection (dpi). The areas delineated by white rectangles in the top panel are shown at higher magnification in the bottom panels. Scale bars represent as indicated. Pinwheel (pw), starch grain (SG).

**Figure 5.**
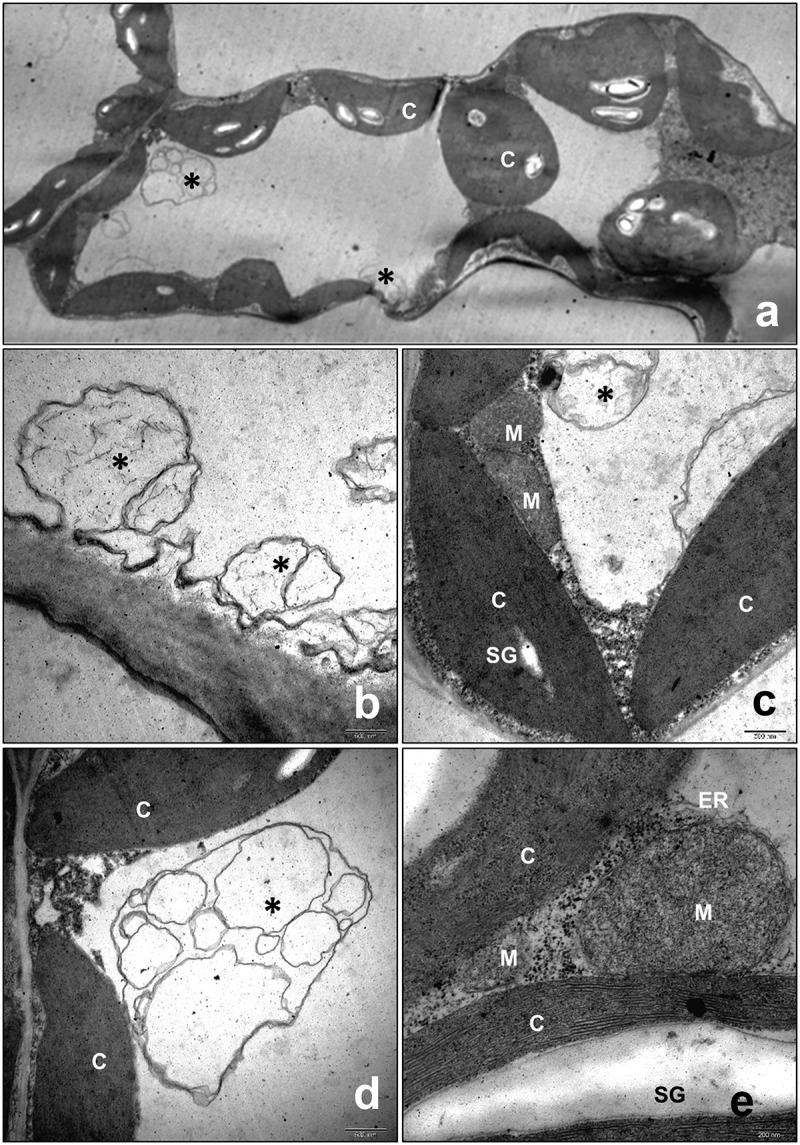
Transmission electron micrographs of autophagosomes in healthy leaves of *Nicotiana benthamiana*. The 8^th^ leaf of an un-infected 9 dpi healthy tobacco plant was observed. The areas delineated by white rectangles in the top panels are shown at higher magnification in the bottom panels. Asterisks (*) indicate autophagosomes. chloroplast (C), mitochondria (M), starch grain (SG), endoplasmic reticulum (ER).

Because abundant viral coat protein was detected near the vascular bundle of infected plants by immunohistochemistry, the leaf tissues near the vascular bundle were observed for the presence of potyvirus. Ultrastructural analysis revealed a characteristic pinwheel and scroll structure of the virus in infected cells (Figures 3 and 4). The viral components occupied a large part of the cytosol, indicating infection by PVY^O^. The ultrastructure of the infected cells revealed an expanded endoplasmic reticulum (ER) structure that was obstructed by adjacent viral particles. Dilated ER in infected cells is associated with ER stress. These results suggest that ER stress may be induced in virus-infected cells, and that ER stress-associated autophagy may be involved in the pathophysiology of infected plants. Because the ER is a platform for autophagosome formation, dynamic virus association may play a role in providing the components required for the removal of viral particles. Large high-density particles enclosed with multiple layers of membrane structure were found in the autophagosomes observed in the 8^th^ leaf at 9 dpi (Figure 3a). The pinwheel structure was also found in vascular cells next to vascular fibers, suggesting movement of the viral particles through phloem cells (Figure 3b). Autophagosome formation has been shown to be localized at the site of chloroplasts. The autophagosome-associated chloroplast membrane disrupted the internal membrane structure of chloroplasts (Figure 3c). The infected chloroplasts were destroyed and had abnormal internal structures. Autophagosome formation was prominent in infected cells and was observed to a small extent in cells with minor infection. In un-infected normal cells, the shape of the organelles was constant, and transparent autophagosomes were observed in the cells (Figure 5).

### 3.7. Expression of autophagy-related proteins in virus-infected plants

**Figure 6.**
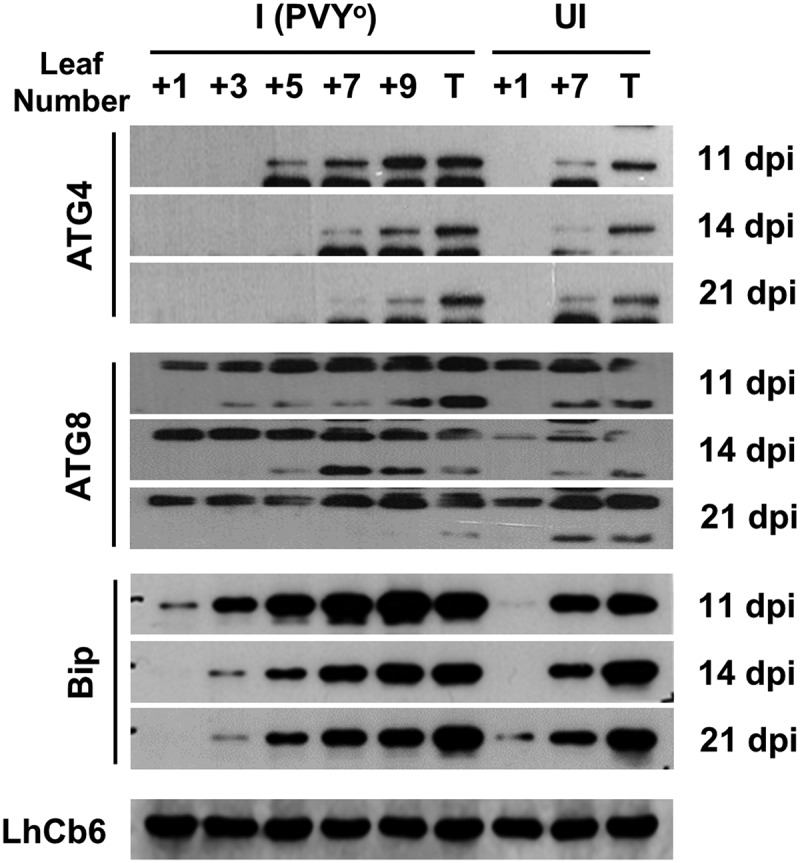
Effect of potato virus Y (PVY) on autophagy-related proteins in systemic leaves of *Nicotiana benthamiana* infected with potato virus Y-O (PVY^O^). The numbers represent the order of systemic leaves above the infected leaf at indicated time {days post-infection (dpi)}. ATG4, ATG8, and BIP protein extracts were prepared from the leaves for western blot analysis to determine the levels of protein accumulation. I, leaves infected with PVY^O^; UI, un-infected healthy leaves as control.

As considerable autophagosome formation was observed with viral structures, the expression levels of the proteins involved in the formation of autophagosomes were examined in infected tobacco plants. In the autophagy pathway, a double-membrane vesicle called an autophagosome encloses intracellular cargo to deliver them into the vacuole. In this experiment, the induction of ATG4 was detected in the upper part of the infected plants, which is the same location as that of viral protein accumulation. However, ATG4 and ATG8 expression levels changed over time. When viral symptoms appeared in the youngest leaf, the expression of ATG4 decreased, suggesting suppression of the autophagy pathway during disease development (Figure 6). In the case of ATG8, constitutive expression was observed in tobacco leaves regardless of viral infection. However, lipidated ATG8 accumulated in young leaves and the level decreased after disease development at 14 dpi (Figure 6). The deposition of ATG4 was then examined in vascular bundles, which are known to be the virus path. Following viral infection, a massive accumulation of ATG4 was observed in the cells around the vascular tissues of the infected plants (Supplementary Fig. S4). These results suggest that ATG4 proteins colocalized with PVY^O^ and may be involved in autophagosome formation in the presence of viral particles. No immunostaining was observed in control sections incubated with preimmune serum (data not shown). In addition, the binding immunoglobulin protein (BiP), an ER stress marker, binds to misfolded proteins to remove abnormal proteins. Protein degradation is critical for the modulation of numerous biological processes under stressful conditions. Upon virus infection, an intense band of BiP was detected in systemic leaves from the upper parts of plants. However, over time, the expression decreased slightly, especially in the lower parts of the plants (Figure 6).

### 3.8. Expression pattern of RNA suppression-related genes

**Figure 7.**
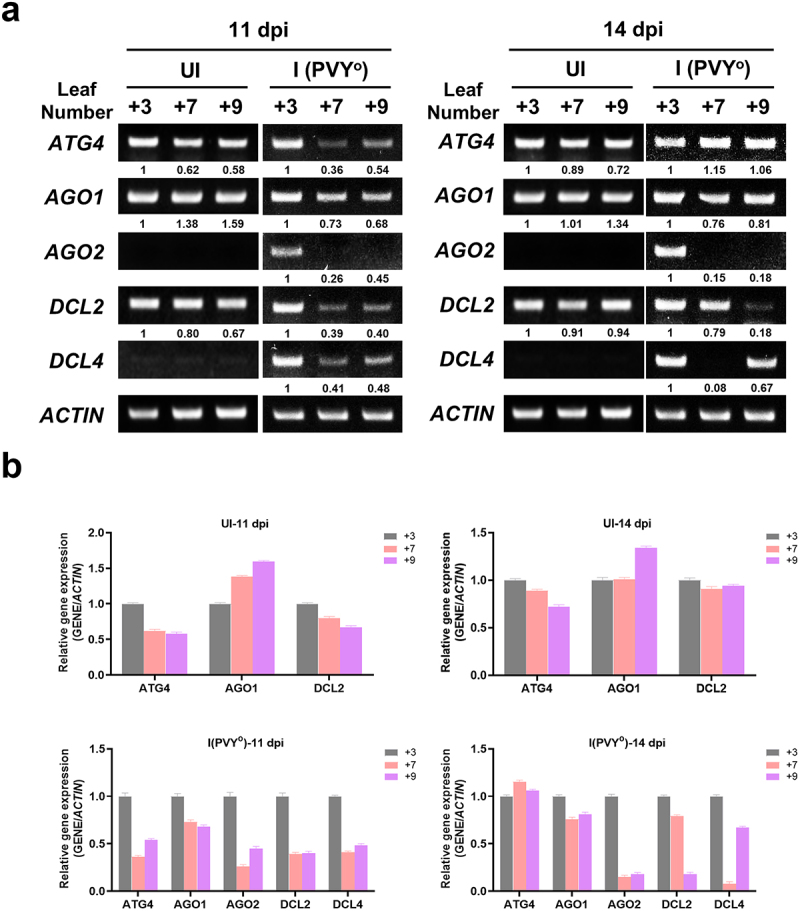
RT-PCR analysis of viral defense related genes in the leaves of *Nicotiana benthamiana* infected with potato virus Y-O (PVY^O^). RNA extracted in 11 and 14 days post-infection (dpi) plants leaf. The leaf number is indicated above the inoculated leaf (3, 7 and 9). (A) autophagy-related genes 4 and 8, *ATG4*, *ATG8*; argonaute 1 and 2, *AGO1*, *AGO2*; dicer-like protein 2, 4, *DCL2* and *DCL4*; *ACTIN* was used as the loading control. I, leaves infected with PVY^O^; UI, un-infected healthy leaves as control. (b) the graph shows the quantitative values for (a).

RNA silencing is a ubiquitous defense mechanism activated by non-coding or dsRNAs. In response to viral invasion, plants can recognize the dsRNA of the virus and have evolved an RNA silencing pathway as a defense mechanism against viruses. Viral dsRNAs are cleaved by dicer-like (DCL) enzymes into small interfering RNAs (siRNAs) that guide the target viral RNA. The target RNA is then degraded by argonaute (AGO) proteins. Thus, siRNAs are likely mobile signals that spread the defense response to prevent viral accumulation in systemic leaves. In this study, the expression levels of genes involved in viral RNA suppression in infected plants were determined in the 3^rd^, 7^th^, and 9^th^ leaves of infected plants at 11 and 14 dpi, and the expression levels of *AGO1*, *AGO2*, *DCL2*, *DCL4*, and *ATG4* were determined (Figure 7(a,b)). The results showed that the expression level of *AGO1* decreased from the 7^th^ leaf of 11 dpi plants compared to that of 14 dpi plants. Unlike the control, the expression of *AGO2* was observed in the 3^rd^ leaf at both 11 and 14 dpi. The expression of *DCL2* was clearly decreased in the 9^th^ leaves of 14 dpi plants compared to that in 11 dpi plants. The expression level of *DCL4* was weak, except in the 3^rd^ leaf at 11 dpi. In the case of *ATG4*, 11 dpi showed a weak overall expression. The results indicate that the enhanced expression of RNA silencing-related genes by PVY infection was compromised in newly growing young leaves, suggesting suppression of RNA silencing during symptom development in the upper leaves.

### 3.9. Expression levels of pathogenesis-related (PR) protein 3 and chlorophyll binding protein in infected plants

Plants respond to phytopathogen invasion with coordinated gene expression. Various genes are induced both at the site of infection and in the distal parts of the plant, leading to the development of the hypersensitive reaction and systemic acquired resistance.^21,22^ The expression levels of several genes are often used as indices of plant resistance to pathogen infections. For example, pathogenesis-related PR genes are induced early, at high levels, during incompatible host-pathogen interactions than during compatible interactions.^23^ Pathogenesis-related proteins do not usually accumulate in healthy plants but are induced by pathogen infection or related stresses and improve the defensive capacity of plants.^24^ In this study, the expression levels of PR protein 3 (PR3) were determined in infected plants at 11, 14, and 21 dpi. At 14 dpi, PR3 expression was detected in the leaves of infected plants (Supplementary Fig. S5). The expression level was significantly higher in the upper parts of the plants at 14 dpi than at 11 dpi. However, the expression levels decreased again at 21 dpi. In addition, at 11, 14, and 21 dpi, the infected plants were examined to clarify chlorophyll-binding protein changes due to the effects of the virus. In the case of LhCb, no significant change was observed over time after infection.

## 4. Discussion

Potato virus Y infection in tobacco causes leaf drop, leaf crinkle, and leaf necrosis, resulting in the inhibition of plant growth. In this study, we observed that the virus migrated directly to the apex through the vascular bundle system and spread. Viruses detected in newly grown leaves appear to be closely related to plant growth. The PVY then multiplied over time from the top of the plant and descended to the lower leaves after developing severe symptoms.

Reactive oxygen species (ROS) play an essential role in plant defense mechanisms. The accumulation of ROS in cells can lead to cell death.^20^ In the current study, ROS production in tobacco increased rapidly at 3 h post infection in response to viral infection. ROS levels remained high for several days in the upper parts of the plants and decreased in the lower leaves. In addition, the expression levels of enzymes that remove ROS from cells were significantly higher in the infected group than in the control group. Even after 7 dpi, the intracellular levels of APX acting on ROS scavenging were high, especially in the chloroplasts.^25^ This phenomenon rapidly removes ROS to reduce intracellular damage. The strongest response was observed in the upper leaves at 11 dpi. The high expression in the upper leaves of infected plants appears to be related to viral accumulation. The upper leaves were continuously damaged by the virus, and mosaic symptoms were observed at 14 dpi combined with decreased expression of antioxidant enzymes.

In infected plants, potyviruses are distributed in plant organelles such as the vacuole, cytosol, chloroplast, and ER.^26–29^ Transmission electron microscopy observations of plant organs indicated that PVY^O^ is located in the ER, chloroplasts, cytoplasm, and vascular bundle. Endoplasmic reticulum stress activates autophagy in mammalian and plant cells. The main causes of ER stress are ROS generation and accumulation of misfolded proteins.^30,31^ BiP is an ER protein that reacts with misfolded proteins and induces the degradation of abnormal proteins. However, BiP is involved in autophagy and is also associated with viral infections.^32,33^ The expression level of BiP increased at 11 dpi and then decreased over time. It is also associated with ATG8, which is involved in autophagosome formation. At 11 dpi, ATG8 is cleaved by ATG4 to produce large amounts of ATG8-phosphatidylethanolamine. However, the ATG8-phosphatidylethanolamine significantly decreased after the appearance of mosaic symptoms. Expression of the Cys protease ATG4 also decreased over time. This suggests that the more severe the infection, the greater is the inhibition of autophagic function. Consequently, the defense mechanisms of virus-infected plants are suppressed by the virus over time. In addition, as the expression of ATG4 was confirmed around the vascular bundle in plants, it seems that the movement of the virus is closely related to autophagy.

Viral RNA silencing is a major defense mechanism of the host against plant viruses. Viral RNA silencing is mediated by host Dicer-like protein (DCL) and AGO proteins. DCL recognizes viral dsRNA and cuts it into short sections of 21–23 nucleotides. Nucleotides bind to viral ssRNA via AGO to form an RNA-induced silencing complex, which inhibits the transcription of viral RNA or degrades it.^34–36^ We investigated the expression of *DCL2*, *DCL4*, *AGO1*, and *AGO2* in PVY^O^-infected plants. *AGO1* and *DCL2* genes did not show significant differences in expression in un-infected plants, but decreased at 11 dpi, the stage before mosaic symptoms appeared in PVY^o^-infected plants. Pathogenesis-related genes encode various antifungal proteins, including glucanase and chitinase. However, the response of PR proteins to viral infection has not yet been reported.^37,38^ The chitinase PR3 showed the highest expression at 14 dpi, when mosaic symptoms appeared. These results suggest that the PR3 protein acts as a defense mechanism in infected plants.

The results of this study suggest the physiological responses and defense mechanisms of PVY^O^ infected plants. In particular, the removal of ROS, activation of autophagy, and RNA silencing are actively induced before 14 dpi, or when the symptoms manifest prominently. However, after 14 dpi and the onset of symptoms, the defense mechanisms of the infected plants were reduced. PVY^o^ migrated to other leaves through the phloem, and many virus particles were detected in the upper leaves where metabolism was most active. In the early response to viral infection, various antioxidant enzymes are expressed to remove ROS and starch grains accumulate. In addition, autophagy was rapidly activated, but activity decreased over time, and defense ability significantly decreased after the appearance of symptoms. In conclusion, we established how PVY proliferates and migrates in plants, as well as the process of plant resistance to viruses and physiological responses after mosaicism. However, further studies related to the viral defense mechanism (RNA silencing) after the onset of mosaic symptoms are needed.

## Supplementary Material

Supplemental Material
