# Supplementary material for: Coordinated recruitment of conserved defense-signaling pathways in PVYO-Infected Nicotiana benthamiana
Source: Plant Signal Behav. 2023 Sep 1;18(1):2252972. doi: 10.1080/15592324.2023.2252972 (PMC10478736; doi:10.1080/15592324.2023.2252972)
Supplement: Supplemental Material [file KPSB_A_2252972_SM3182.docx]

**Supplementary Table 1. *Nicotiana benthamiana* gene-specific primers used in RT-PCR**

| Gene | Primers (5' to 3') | |
| --- | --- | --- |
| ATG4 | Forward: | GGTGCTCCATTGTTTACGATAAC (20 mer) |
|  | Reverse: | GCAATCAAGTAGAACGACCAGC (22 mer) |
| AGO1 | Forward: | TATGGGAAGGAGCACGCGAG (20 mer) |
|  | Reverse: | AGCCAAGCCAAGGTGTGACA (20 mer) |
| AGO2 | Forward: | TCCAACTTCCACGACCCCAC (20 mer) |
|  | Reverse: | TGCTGTCACCACCACCAAGT (20 mer) |
| DCL2 | Forward: | CGTGAGAATGGCGTGGCGTA (20 mer) |
|  | Reverse: | AGGGCAGAAACCAAAACATACGA (23 mer) |
| DCL4 | Forward: | CTGAGGACGCAGCAGAAGG (19 mer) |
|  | Reverse: | AGACCCCTTTATCACTCCGTACT (23 mer) |
| ACTIN | Forward: | CAATGAGCTTCGTGTTGCGCC (21 mer) |
|  | Reverse: | CAAACTATCAGTGAGGTCACGG (22 mer) |

**Supplementary Table 2. Primary antibody information used in western blot**

| **Antibody name** | **Manufacturer** | **Host** | **Dilution rate** |
| --- | --- | --- | --- |
| APX | Agrisera | Rabbit | 1:2,000 |
| ATG4 | Agrisera | Rabbit | 1:2,500 |
| ATG8 | Agrisera | Rabbit | 1:1,000 |
| BiP | Agrisera | Rabbit | 1:1,500 |
| CAT | Agrisera | Rabbit | 1:2,000 |
| Cu/ZnSOD (CSD2) | Agrisera | Rabbit | 1:2,000 |
| FeSOD | Agrisera | Rabbit | 1:2,000 |
| LhCa1 | Agrisera | Rabbit | 1:5,000 |
| LhCa2 | Agrisera | Rabbit | 1:5,000 |
| LhCb6 | Agrisera | Rabbit | 1:5,000 |
| MnSOD | Agrisera | Rabbit | 1:2,000 |
| PR3 | Agrisera | Rabbit | 1:3,000 |
| PVY-VN | Seoul Women's University | Rabbit | 1:2,000 |

**Supplementary Fig. S1**


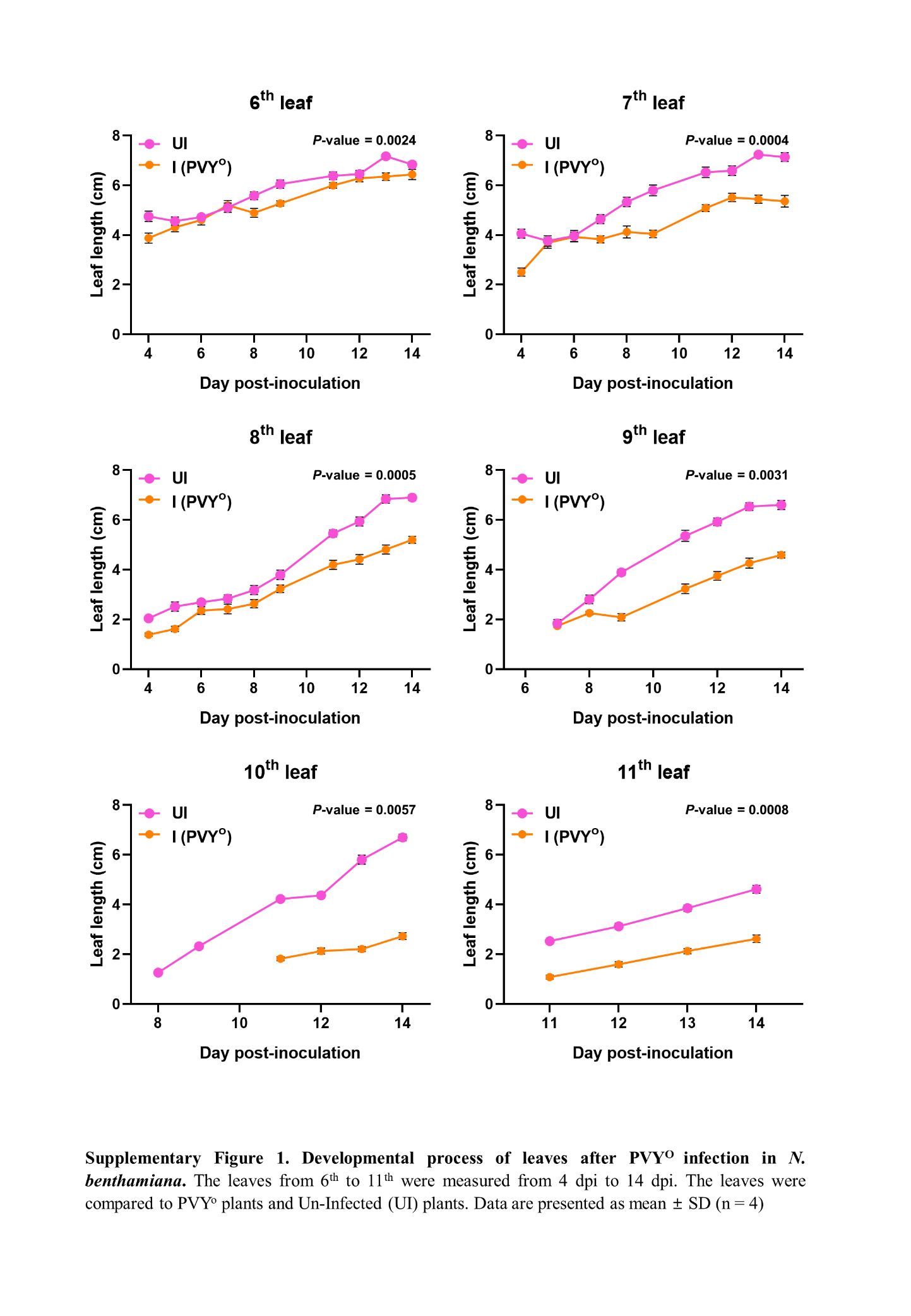


**Supplementary Fig. S1. Developmental process of leaves after PVY^O^ infection in *N. benthamiana*.** The leaves from 6^th^ to 11^th^ were measured from 4 dpi to 14 dpi. The leaves were compared to PVY^o^ plants and Un-Infected (UI) plants. Data are presented as mean ± SD (n = 4)

**Supplementary Fig. S2**


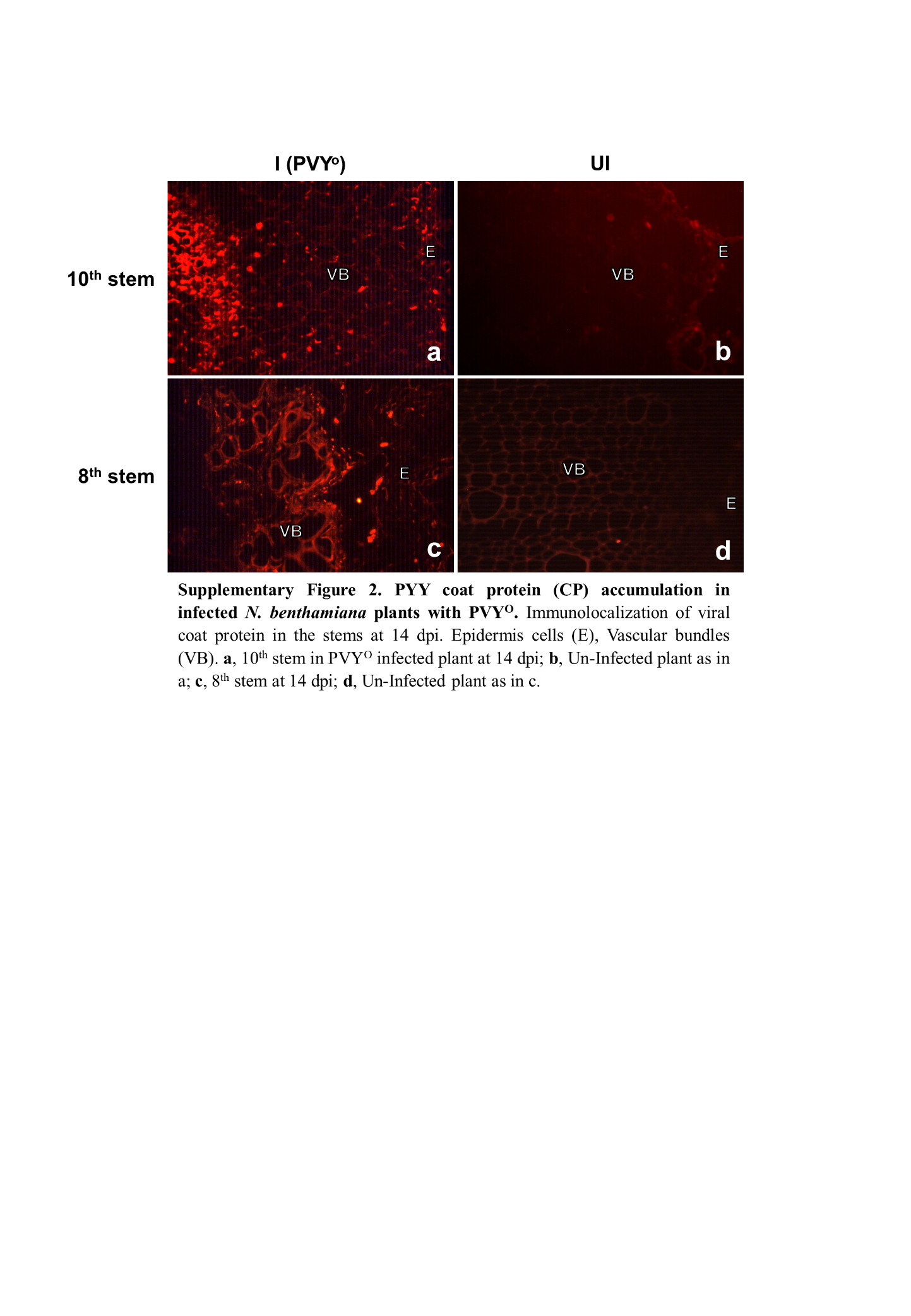


**Supplementary Fig. S2. PYY coat protein (CP) accumulation in infected *N. benthamiana* plants with PVY^O^.** Immunolocalization of viral coat protein in the stems at 14 dpi. Epidermis cells (E), Vascular bundles (VB). **a**, 10^th^ stem in PVY^O^ infected plant at 14 dpi; **b**, Un-Infected plant as in a; **c**, 8^th^ stem at 14 dpi; **d**, Un-Infected plant as in c.

**Supplementary Fig. S3**


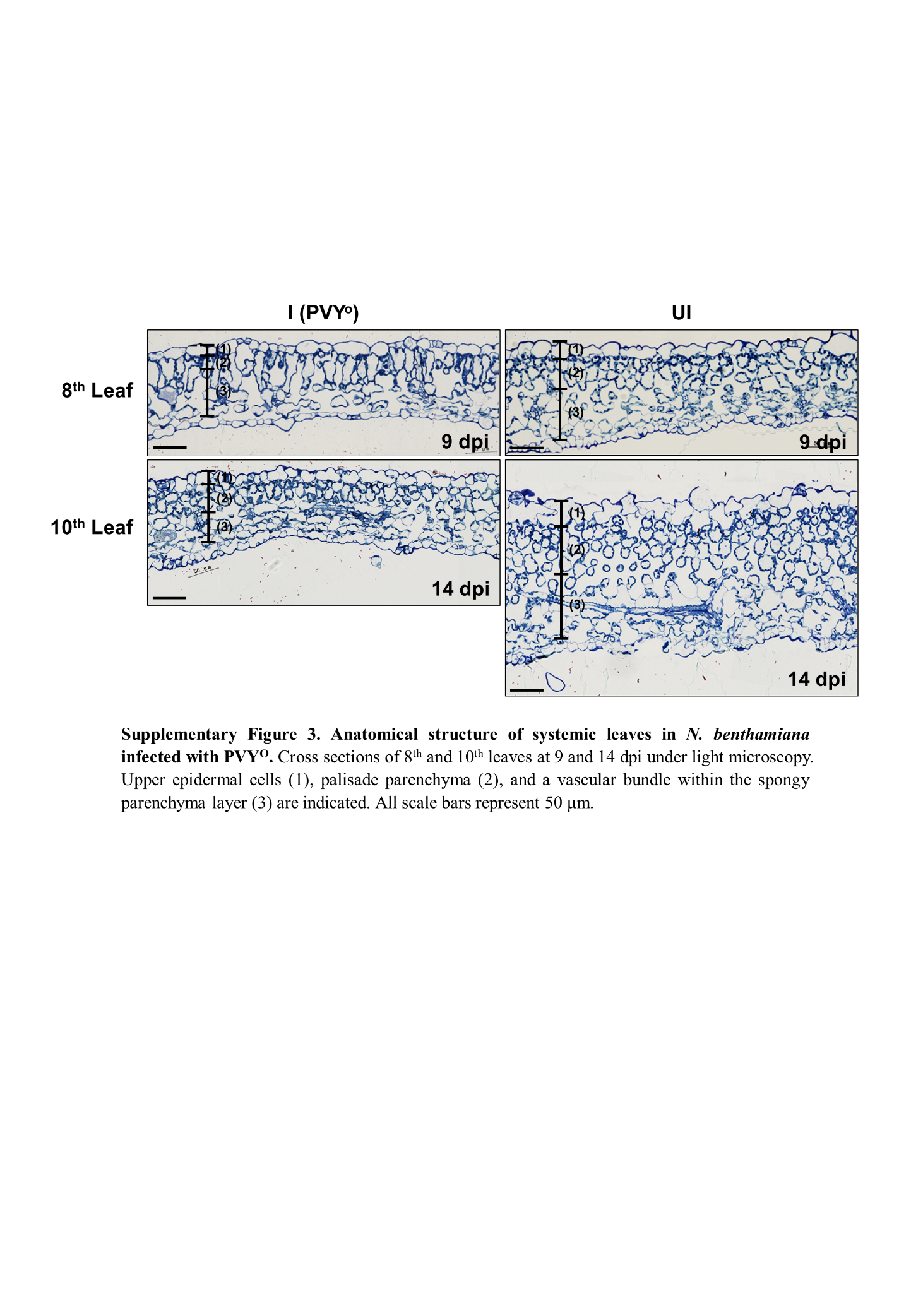


**Supplementary Fig. S3. Anatomical structure of systemic leaves in *N. benthamiana* infected with PVY^O^.** Cross sections of 8^th^ and 10^th^ leaves at 9 and 14 dpi under light microscopy. Upper epidermal cells (1), palisade parenchyma (2), and a vascular bundle within the spongy parenchyma layer (3) are indicated. All scale bars represent 50 μm.

**Supplementary Fig. S4**


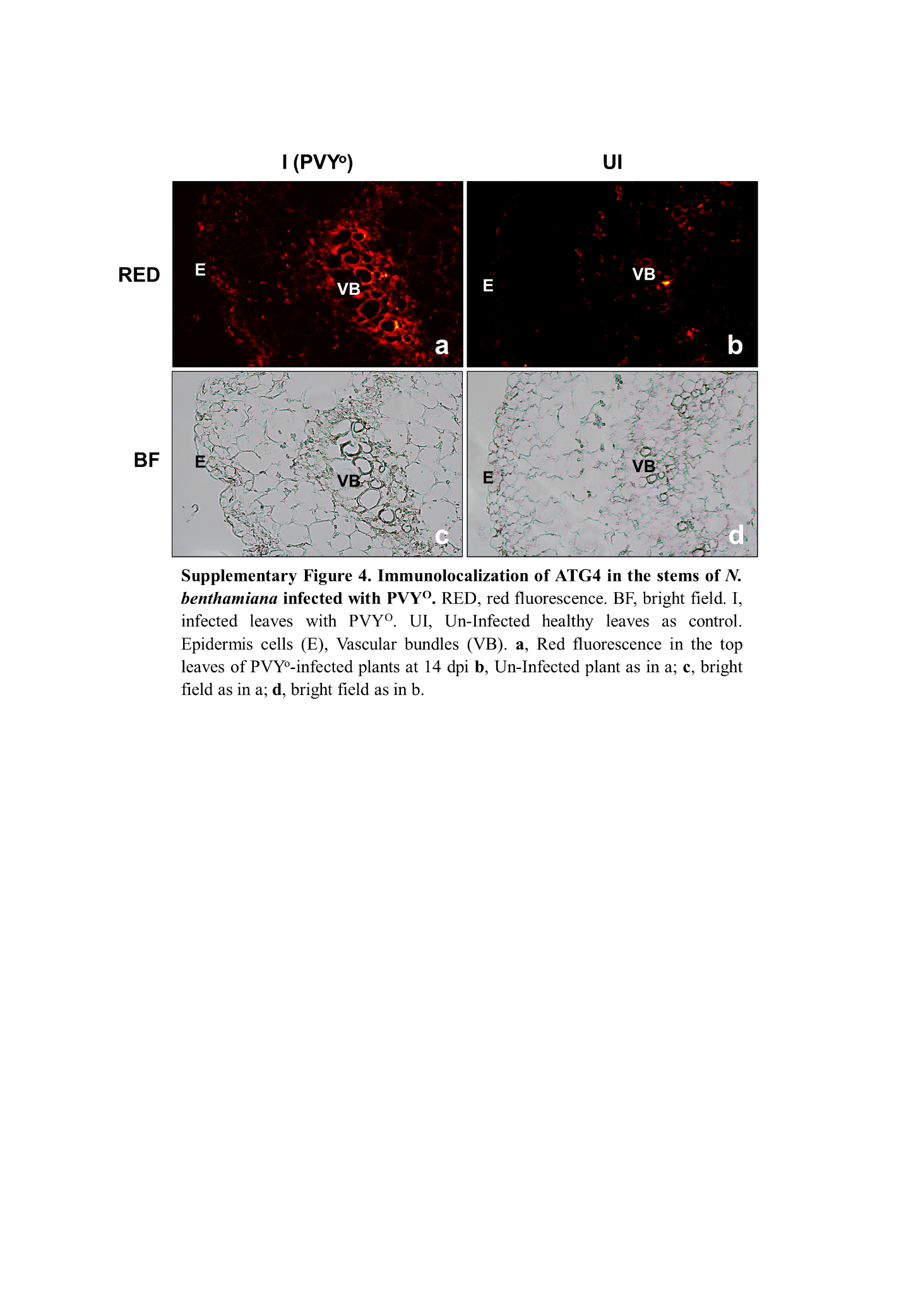


**Supplementary Fig. S4. Immunolocalization of ATG4 in the stems of *N. benthamiana* infected with PVY^O^.** RED, red fluorescence. BF, bright field. I, infected leaves with PVY^O^. UI, Un-Infected healthy leaves as control. Epidermis cells (E), Vascular bundles (VB). **a**, Red fluorescence in the top leaves of PVY^o^-infected plants at 14 dpi **b**, Un-Infected plant as in a; **c**, bright field as in a; **d**, bright field as in b.

**Supplementary Fig. S5**


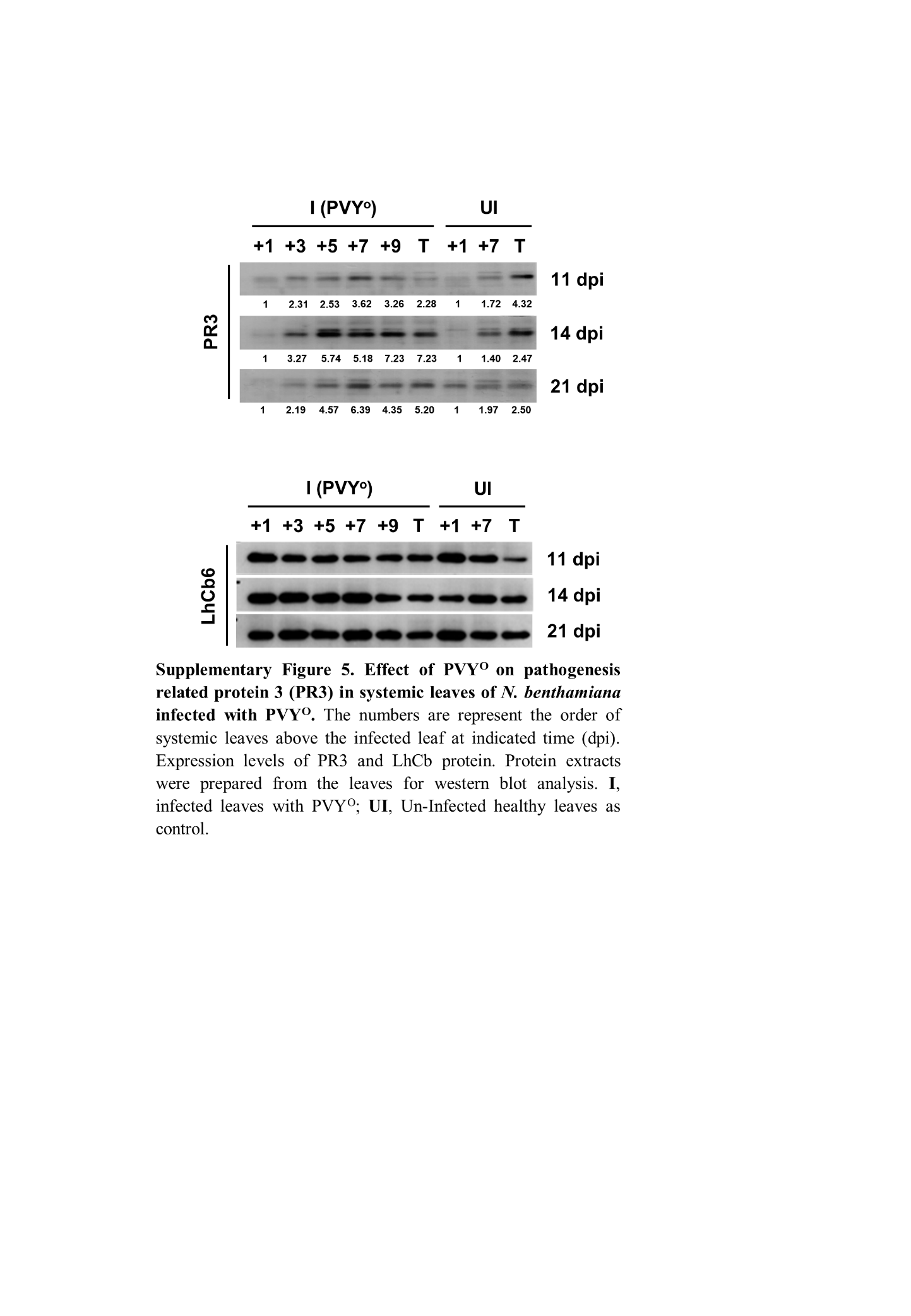


**Supplementary Fig. S5. Effect of PVY^O^ on pathogenesis related protein 3 (PR3) in systemic leaves of *N. benthamiana* infected with PVY^O^.** The numbers are represent the order of systemic leaves above the infected leaf at indicated time (dpi). Expression levels of PR3 and LhCb protein. Protein extracts were prepared from the leaves for western blot analysis. **I**, infected leaves with PVY^O^; **UI**, Un-Infected healthy leaves as control.
